# Comparative proteomics reveals human pluripotent stem cell-derived limbal epithelial stem cells are similar to native ocular surface epithelial cells

**DOI:** 10.1038/srep14684

**Published:** 2015-10-01

**Authors:** Alexandra Mikhailova, Antti Jylhä, Jochen Rieck, Janika Nättinen, Tanja Ilmarinen, Zoltán Veréb, Ulla Aapola, Roger Beuerman, Goran Petrovski, Hannu Uusitalo, Heli Skottman

**Affiliations:** 1 BioMediTech, University of Tampere, Finland; 2 Department of Ophthalmology, School of Medicine, University of Tampere, Finland; 3 Stem Cells and Eye Research Laboratory, Department of Ophthalmology, Faculty of Medicine, University of Szeged, Hungary; 4 Singapore Eye Research Institute and School of Medicine, Singapore; 5 Tampere University Hospital Eye Center, University of Tampere, Finland

## Abstract

Limbal epithelial stem cells (LESCs) are tissue-specific stem cells responsible for renewing the corneal epithelium. Acute trauma or chronic disease affecting LESCs may disrupt corneal epithelial renewal, causing vision threatening and painful ocular surface disorders, collectively referred to as LESC deficiency (LESCD). These disorders cannot be treated with traditional corneal transplantation and therefore alternative cell sources for successful cell-based therapy are needed. LESCs derived from human pluripotent stem cells (hPSCs) are a prospective source for ocular surface reconstruction, yet critical evaluation of these cells is crucial before considering clinical applications. In order to quantitatively evaluate hPSC-derived LESCs, we compared protein expression in native human corneal cells to that in hPSC-derived LESCs using isobaric tag for relative and absolute quantitation (iTRAQ) technology. We identified 860 unique proteins present in all samples, including proteins involved in cell cycling, proliferation, differentiation and apoptosis, various LESC niche components, and limbal and corneal epithelial markers. Protein expression profiles were nearly identical in LESCs derived from two different hPSC lines, indicating that the differentiation protocol is reproducible, yielding homogeneous cell populations. Their protein expression profile suggests that hPSC-derived LESCs are similar to the human ocular surface epithelial cells, and possess LESC-like characteristics.

Corneal epithelium, the outermost layer of the transparent and avascular cornea, is a rapidly-regenerating stratified squamous epithelium. Its integrity and maintenance are essential for corneal transparency and normal vision. Limbal epithelial stem cells (LESCs) are a type of tissue-specific stem cells located at the corneoscleral junction within niche regions of the palisades of Vogt^1^,^2^. These stem cells are crucial for maintaining the ocular surface in two ways: first, they constantly renew the corneal epithelium, as the topmost layers are shed off into the tear film; and second, they serve as a physical barrier between the corneal and conjunctival epithelia^3^,^4^. Like other tissue-specific stem cells, LESCs are thought to be slow cycling, yet with a potential for self-renewal, rapid proliferation, and differentiation in response to appropriate stimuli^5^,^6^. LESCs give rise to transient amplifying cells (TACs) that have a higher capacity for proliferation and differentiation. TACs help preserve the normal homeostasis of the corneal epithelium by migrating apically towards the center of the cornea and replacing the lost corneal epithelial cells (CECs). Acute trauma or chronic disease affecting LESCs may cause a disruption in this homeostasis and allow the neighboring conjunctival epithelial cells, along with blood vessels, to migrate over the ocular surface^7^. Such ocular surface disorders are collectively referred to as LESC deficiency (LESCD), and they are difficult to treat with conventional corneal transplantation, as corneal grafts do not replace the damaged limbus^8^.

Various strategies have been investigated to facilitate the reconstruction of damaged ocular surface, such as transplantation of autologous or allogeneic limbal tissue, or more recently cultivated limbal epithelial transplantation (CLET), where a small amount of autologous or allogeneic LESCs is expanded *in vitro* before being transplanted to the ocular surface^4^,^5^,^9^,^10^. In fact, Holoclar, the first advanced therapy medicinal product containing autologous LESCs was recently granted conditional approval by the European Medicines Agency. Despite the generally promising results of CLET, it is limited by variation in long-term success rates, use of xenogeneic and undefined culture components, and scarcity of donor tissue^11^,^12^. This is especially important in bilateral LESCD cases, where autologous tissue is unsuitable for CLET, and alternative solutions are needed. Human pluripotent stem cells (hPSCs), namely human embryonic stem cells (hESCs) and human induced pluripotent stem cells (hiPSCs), are readily available in limitless supply, and have a vast differentiation potential. They provide new opportunities for cell-based tissue engineering and drug discovery, and offer novel ways to study human development. Successful differentiation of corneal epithelial lineages has been reported using both hESCs and hiPSCs^13^,^14^,^15^,^16^,^17^. We have previously described an efficient differentiation method from hPSCs towards LESC-like corneal epithelial progenitor cells in feeder-free and serum-free conditions^18^.

Thorough characterization of differentiated cells is an essential step towards clinical applications, as it is important to verify the authenticity of cell populations prior transplantation to the ocular surface. In this study, we compared protein expression in human CECs and limbal epithelial cells (LECs) to that in hESC-derived LESCs (hESC-LESCs) and hiPSC-derived LESCs (hiPSC-LESCs) using isobaric tag for relative and absolute quantitation (iTRAQ) technology. Additionally, protein expression of several putative LESC markers was verified using flow cytometry and immunofluorescence. Recent advances in mass spectrometry (MS) techniques have proven that MS-based approaches with quantitative analyses can contribute to identification of proteins involved in stem cell proliferation and differentiation^19^,^20^,^21^. The main advantage of using iTRAQ proteomics is that it allows multiplexing with four or eight different isobaric tags. Both 4-plex and 8-plex iTRAQ methods have been utilized to analyze and study protein expression during differentiation of mouse or human PSCs^22^,^23^,^24^. We used 4-plex iTRAQ to enable four samples to be compared directly within the same measurement. To our knowledge, this is the first study comparing hPSC-LESCs with their native counterparts using MS-based proteomics.

## Results

**Figure 1:**
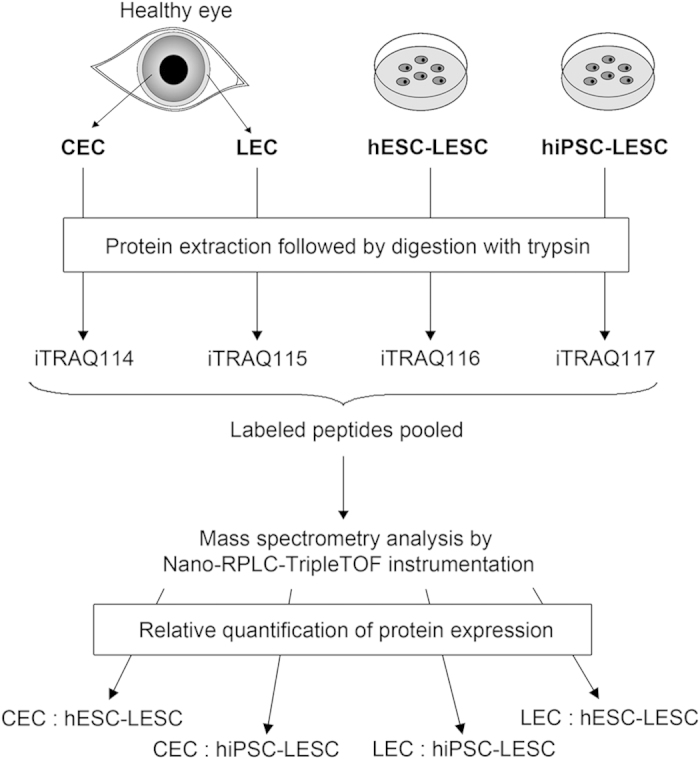
Workflow of the study. Human CECs were collected from the surface of the central cornea, while LECs were collected from the surface of the limbus (1–2 mm wide zone between the cornea and the sclera) of three cadaveric donors. Three biological replicates of hESC-LESCs and hiPSC-LESCs were obtained using a directed differentiation method. Total protein was extracted from all samples and digested with trypsin. Peptides were labeled with iTRAQ 4-plex reagents and analyzed using Nano-RPLC-Triple TOF instrumentation to obtain four separate comparisons from each set of biological replicates. Comparative proteomics using the iTRAQ technology is only capable of identifying proteins that are expressed in all analyzed samples.

In this study, native human CECs and LECs were compared to hESC-LESCs and hiPSC-LESCs using iTRAQ proteomics (Fig. 1). Central corneal (CEC) and limbal (LEC) epithelial samples were collected by positional scraping of cells directly from the surface of cadaver eyes (three separate donors), without additional purification. Differentiation of hESCs and hiPSCs towards LESC-like cells was carried out with three biological replicates.

### Distribution of identified proteins

**Figure 2:**
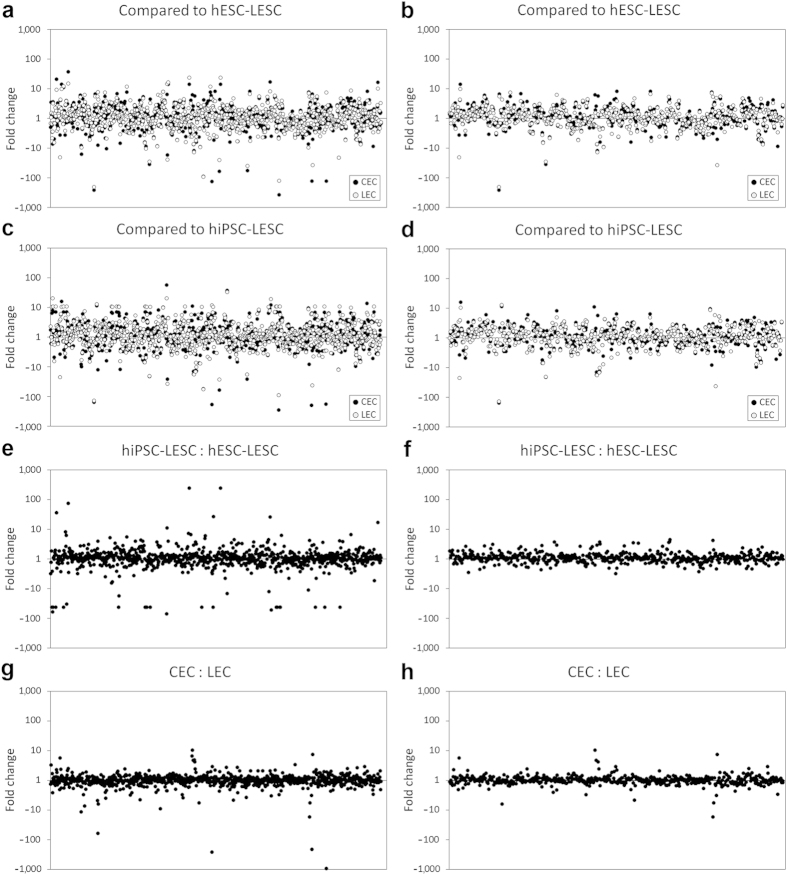
Overview of protein expression profiles. Only the proteins identified in at least two of the three biological replicates were considered reliable, and were kept for further analyses. (**a**,**c**,**e**,**g**) unfiltered results; (**b**,**d**,**f**,**h**) filtered results. Each dot represents a single identified protein, presented as mean fold changes on a logarithmic scale, where *y* = 1 signifies equal protein expression. CECs (black dots) and LECs (gray dots) compared to (**a**,**b**) hESC-LESCs and (**c**,**d**) hiPSC-LESCs have very similar expression profiles. (**e**,**f**) Comparison of hiPSC-LESCs and hESC-LESCs shows that they have consistent protein expression profiles. (**g**,**h**) Comparison of CECs and LECs reveals a high level of similarity in their expression profiles. Complete list of identified proteins is provided in Supplementary Table S1.

**Figure 3:**
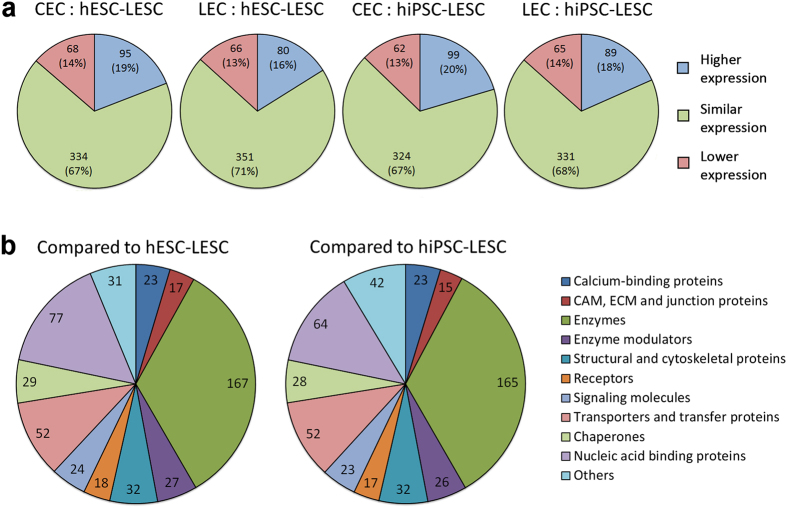
Distribution of identified proteins. The filtered data sets were grouped according to (**a**) protein expression differences in each of the four comparisons, or (**b**) protein class in human CECs and LECs compared to hESC-LESCs or hiPSC-LESCs. Differences in protein expression greater than 2-fold were considered as biologically significant (higher expression: greater than 2-fold; similar expression: between -2 and 2-fold; lower expression: less than -2-fold). Complete list of identified proteins is provided in Supplementary Table S1.

A total of 860 unique proteins expressed in all four samples were identified (Fig. 2). The complete list of identified proteins and their respective expression levels is provided in Supplementary Table S1. For a more detailed analysis, proteins that were detected in only one of the biological replicates, as well as obsolete protein IDs were discarded. After this filtering, 497 proteins were left when CECs and LECs were compared to hESC-LESCs, and 485 proteins when compared to hiPSC-LESCs (Fig. 2). Differences in protein expression greater than 2-fold were considered as over or under-expression. A large portion of the identified proteins were similarly expressed (between -2 and 2-fold) in all samples (Fig. 3a). With the help of PANTHER classification, the filtered set of proteins was categorized by their molecular function (Fig. 3b). The largest protein class in the data set was enzymes, containing the following sub-groups: hydrolases, isomerases, kinases, ligases, lyases, oxidoreductases, phosphatases, proteases and transferases. Some of the most interesting protein classes in the case of corneal and limbal epithelium are adhesion and junction proteins, structural and cytoskeletal proteins and signaling molecules. These protein classes are commonly involved in maintaining stem cell or TAC behavior and the niche microenvironment. Therefore, these groups of proteins and the differences in their expression were examined in more detail.

### Proteins related to stem cell or TAC behavior

**Figure 4:**
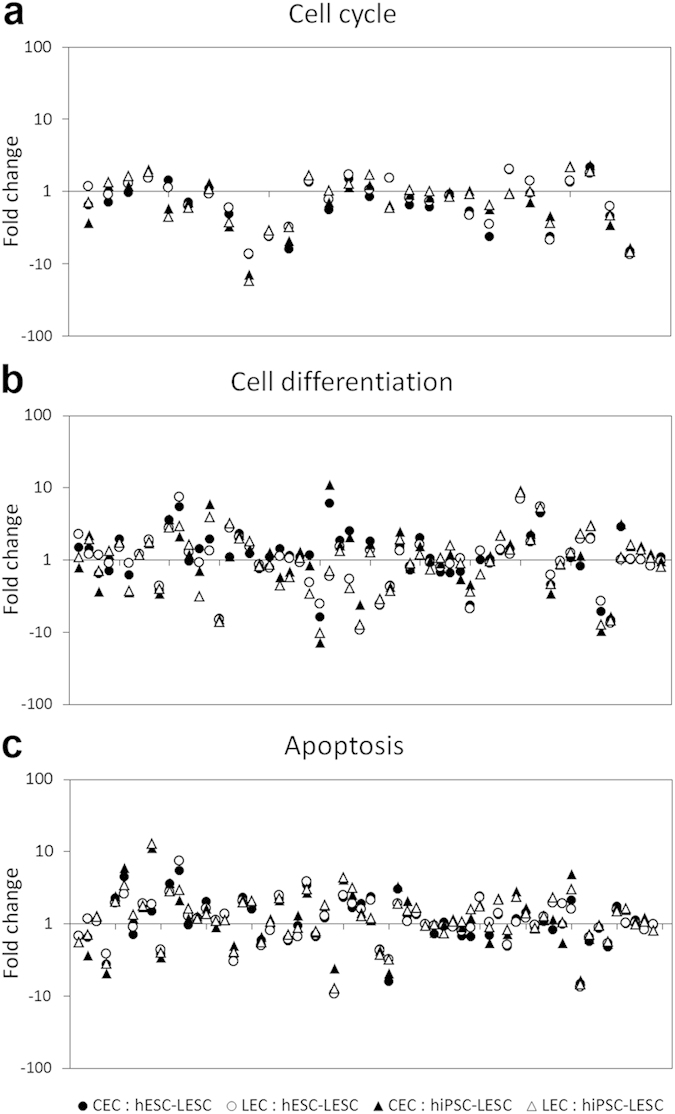
Proteins involved in cell cycling, differentiation and apoptosis. Expression differences of (**a**) cell cycle proteins, (**b**) proteins involved in cell differentiation, or (**c**) apoptosis, in native CECs (black markers) and LECs (gray markers) as compared to hESC-LESCs and hiPSC-LESCs. Each marker represents a single unique protein, presented as mean fold changes on a logarithmic scale, where *y* = 1 signifies equal protein expression. Lists of proteins and their expression in numeric values are provided in Supplementary Table S2.

All stem cells, including LESCs, are generally quiescent and slow cycling, while TACs undergo active cell cycling, proliferation, differentiation and apoptosis. In this study, 31 proteins involved in regulating cell proliferation were identified in all samples: 14 positive regulators, 13 negative regulators, and 4 proteins involved in both processes (Table 1). Furthermore, 59 proteins involved in cell differentiation, 65 proteins involved in apoptosis, and 28 cell cycling proteins were identified (Fig. 4). Most of these proteins were similarly expressed in all samples. Several proteins in these groups, for instance Annexin 1 (ANXA1), galectins 1, 3 and 7 (LGALS1, -3 and -7), and stratifin (SFN), are especially relevant to the maintenance and renewal of the ocular surface.

### Stem cell niche components of the ocular surface

**Figure 5:**
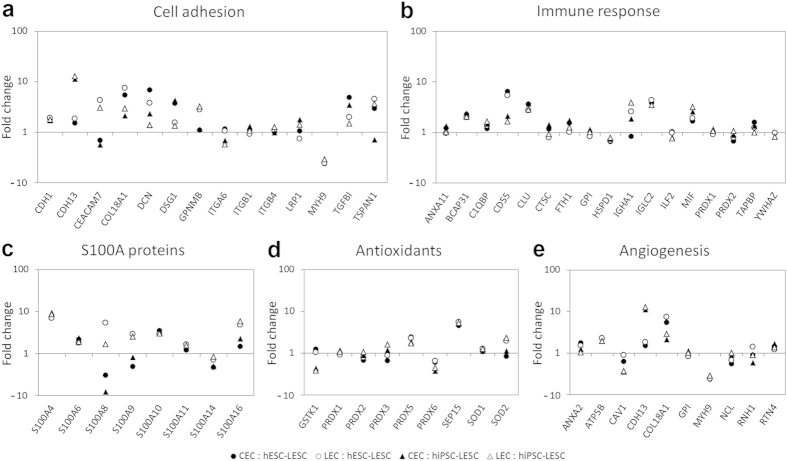
Niche components of the ocular surface. Expression differences of (**a**) cell adhesion proteins, (**b**) proteins involved in immune response, (**c**) S100A proteins, (**d**) antioxidants, and (**e**) angiogenic proteins, in native CECs (black markers) and LECs (gray markers) as compared to hESC-LESCs and hiPSC-LESCs. Each marker represents a single unique protein, presented as mean fold changes on a logarithmic scale, where *y* = 1 signifies equal protein expression. Lists of proteins and their expression in numeric values are provided in Supplementary Table S3.

Niche microenvironment of stem or progenitor cells involves various components such as cell adhesion and junction proteins, calcium-binding S100A proteins, as well as proteins with antioxidant activity or involvement in angiogenesis and immune response, including various growth factors and cytokines secreted by ocular surface epithelial cells. Altogether 14 proteins involved in cell adhesion, junction formation or extracellular matrix (ECM) deposition were identified in this study (Fig. 5a). This group includes several cadherins and integrins, and most of the proteins were similarly expressed in all samples, or mildly overexpressed in CECs and LECs. One clear exception was carcinoembryonic antigen-related cell adhesion molecule 7 (CEACAM7): its expression was higher in LECs and slightly lower in CECs, compared to hPSC-LESCs. Additionally, cadherin 13 (CDH13) was overexpressed in CECs and LECs compared to hiPSC-LESCs. All four samples were found to express 17 proteins involved in immune response (Fig. 5b). Most of these proteins were similarly expressed in all samples, with the following exceptions: immunoglobulin heavy constant alpha 1 (IGHA1) was overexpressed in LECs, CD55, clusterin (CLU) and immunoglobulin lambda constant 2 (IGLC2) were overexpressed in CECs and LECs, while macrophage migration inhibitory factor (MIF) was only overexpressed compared to hiPSC-LESCs. S100A proteins form the largest family of calcium-binding proteins, and are known to be expressed at the limbus. Eight members of this protein family were identified in the analyzed data set (Fig. 5c). When compared to hPSC-LESCs, proteins S100A8 and -A9 were found to be expressed at lower levels in CECs, and at higher levels in LECs. The remaining proteins were either mildly overexpressed in CECs and LECs, or similarly expressed in all samples. Moreover, 9 proteins with antioxidant properties and 10 proteins with a role in angiogenesis were identified, most of which were similarly expressed in all samples (Fig. 5d,e).

### Corneal and limbal markers

**Figure 6:**
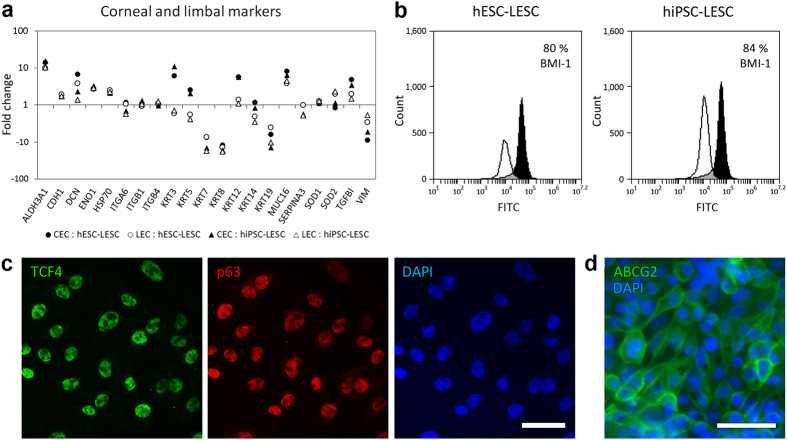
Corneal and limbal markers. (**a**) Expression differences in native CECs (black markers) and LECs (gray markers) as compared to hESC-LESCs and hiPSC-LESCs. Each marker represents a single unique protein, presented as mean fold changes on a logarithmic scale, where *y* = 1 signifies equal protein expression. List of proteins and their expression in numeric values are provided in Supplemental Table S4. (**b**) Protein expression of BMI-1 in hPSC-LESCs quantified by flow cytometry. White histograms represent isotype controls, and black histograms represent BMI-1 staining. (**c**) Representative images of TCF4 and p63 protein expression in hiPSC-LESCs, visualized using immunofluorescence. (**d**) Representative image of ABCG2 protein expression in hiPSC-LESCs. Scale bars 50 μm.

A total of 21 proteins important for the structure and function of corneal and limbal epithelial cells were identified using iTRAQ proteomics (Fig. 6a). Six of these proteins are known to be prevalent in the central corneal epithelium: aldehyde dehydrogenase 3, family member 1 (ALDH3A1), decorin (DCN), cytokerains 3 and 12 (KRT3 and KRT12), mucin 16 (MUC16), and transforming growth factor-beta-induced protein (TGFBI). These proteins were found to be expressed at a higher level in CECs than in LECs and hPSC-LESCs. Moreover, KRT3 was expressed at a lower level in LECs than in hPSC-LESCs, and KRT12 was similarly expressed in LECs and hPSC-LESCs. The remaining 15 proteins tend to be more prevalent in native limbal epithelium: cadherin 1 (CDH1), α-enolase (ENO1), heat shock 70 kDA protein 1A/1B (HSP70), integrins α6, β1 and β4 (ITGA6, ITGB1, ITGB4), superoxide dismutase 1 and 2 (SOD1 and SOD2), cytokeratins 5, 7, 8, 14, and 19, SERPINA3, and vimentin (VIM). Of these proteins, KRT7, KRT8, KRT19, and VIM were expressed at lower levels in CECs and LECs compared to hPSC-LESCs. KRT5 expression levels were similar to that of KRT3, and the remaining 11 proteins were either similarly expressed in all samples, or mildly overexpressed in CECs and LECs. Several key LESC markers could not be identified using comparative iTRAQ proteomics, thus their expression in hPSC-LESCs was verified using flow cytometry or immunofluorescence. BMI-1 was expressed in 80% of hESC-LESCs and 84% of hiPSC-LESCs, p63 and TCF4 were co-expressed in most cells, and ABCG2 was localized to the cell membranes (Fig. 6b–d).

## Discussion

Differentiation of hPSCs towards LESC-like cells offers a novel and unlimited cell source for the treatment of severe ocular surface disorders. However, before proceeding further towards clinical applications, it is important to thoroughly characterize the differentiated cell populations. We have previously shown that hPSC-LESCs possess the appropriate cell morphology, express several key LESC markers, and most importantly lack pluripotency markers^18^. Nevertheless, a high-throughput characterization method would be more informative when dealing with hPSC-derived cells. In this study, iTRAQ proteomics were used to compare native corneal and limbal epithelia obtained from cadaveric human donors with hPSC-LESCs differentiated in the absence of serum and feeder cells. A total of 860 unique proteins expressed in all samples were identified, and about 57% of these proteins were present in at least two biological replicates. In general, protein expression levels were very similar in hESC-LESCs and hiPSC-LESCs, meaning that the differentiation protocol is highly reproducible, yielding homogeneous cell populations. Meanwhile, native human CECs and LECs collected from the ocular surface also showed similar protein expression profiles, suggesting they were likely mixed populations of corneal epithelial cells at various stages of maturity, ranging from LESCs to terminally-differentiated cells. The aim of this study was to focus more on overall expression profiles, rather than differences in expression of specific proteins. Here we highlight the most interesting findings regarding stem cell and TAC behavior, limbal niche components and proteins specific to corneal and limbal epithelia.

LESCs, like other tissue-specific stem cells, tend to be metabolically dormant and slow cycling, gradually changing as they mature towards TACs and further into terminally-differentiated CECs^3^. In this study, various proteins involved in cell cycling, differentiation, proliferation and apoptosis were identified. Most proteins were similarly expressed in all samples, and some proteins are known to be involved in more than one process. For example, ANXA1, a calcium- and phospholipid- binding protein, has anti-inflammatory and anti-migratory properties, while being involved in cell differentiation, cell cycling and negative regulation of apoptosis^25^. ANXA1 was previously shown to be four times more prevalent in limbal epithelium compared to corneal epithelium^26^. Here, ANXA1 was indeed expressed at a higher level in LECs than CECs. Furthermore, ANXA1 was expressed at similar levels in LECs and hPSC-LESCs, suggesting that hPSC-LESCs are less mature than CECs. Galectins are a family of β-galactoside-binding proteins implicated in stem cell and TAC behavior by modulating cell-cell and cell-matrix interactions. Three members of this family were identified in our data set: LGALS1, -3 and -7. LGALS1 may regulate apoptosis, cell proliferation and cell differentiation, while LGALS3 is a galactose-specific lectin which binds IgE, and is known to form a complex with ITGA3, ITGB1 and chondroitin sulfate proteoglycan 4 (CSPG4)^27^. LGALS7 is mainly expressed in stratified squamous epithelia and has been shown to play a role in corneal epithelial cell migration and re-epithelialization of corneal wounds^28^. In this study, LGALS1 was found to be expressed at lower levels in CECs and LECs as compared to hPSC-LESCs, LGALS3 was expressed at similar levels in all samples, and LGALS7 was overexpressed in native CECs and LECs. The general similarity in expression of proteins involved in cell cycling, differentiation, proliferation and apoptosis supports our hypothesis that hPSC-LESCs are similar to their native counterparts.

LESCs are known to reside in niche regions of the palisades of Vogt at the corneoscleral junction. There are various niche components crucial for the proper function of LESCs, including ECM components and various cell adhesion proteins such as cadherins and integrins^29^,^30^. Two cadherins (CDH1 and CHD13) and three integrins (ITGA6, ITGB1 and ITGB4) were identified in this study. CDH13 was overexpressed in CECs and LECs compared to hiPSC-LESCs, while the remaining proteins were expressed at similar levels in all samples. CDH1 is important in desmosomal junction formation and stratified epithelium transformation, and was previously found to be slightly upregulated in LESCs compared to CECs at the gene level^31^. Integrin α6/β4 is a receptor for laminin in epithelial cells and it plays a critical structural role in the hemidesmosome^32^. Taken together, the two cadherins and three integrins detected in all the analyzed samples verify the epithelial nature of hPSC-LESCs. TGFBI protein binds to type I, II, and IV collagens and may play an important role in cell-collagen interactions^33^. It is highly expressed in the corneal epithelium and mutations in the *TGFBI* gene are associated with multiple types of corneal dystrophy^34^. This protein was found to be overexpressed in CECs, and similarly expressed in LECs as compared to hPSC-LESCs in our data set. Interestingly, despite the lack of interaction with other cell types and simplified culture environment in which hPSC-LESCs are maintained, the overall levels of cell adhesion protein expression were similar to that in native CECs and LECs.

Another LESC niche component are the S100A proteins, known to be involved in the regulation of many cellular processes such as calcium homeostasis, cytoskeleton organization, stress response, cell motility, proliferation and differentiation^35^. Several S100A proteins, including S100A6, S100A10 and S100A11 bind annexins and are involved in cell membrane organization, ion channel modulation and keratinocyte differentiation^35^. Common ocular surface diseases such as dry eye, pterygium and corneal angiogenesis involve S100 family of proteins, particularly S100A8 and S100A9^36^. Most importantly, S100A4 and A9 proteins have been found to be potent markers of limbal epithelial crypt cells, with a probable involvement in cell differentiation, regulation of growth and cellular structure^37^. S100A8 has also been identified as a putative LESC marker^26^. Here in our study, S100A8 and A9 were expressed at higher levels in LECs, and at lower levels in CECs, as compared to hPSC-LESCs. These results suggest that hESC-LESCs and hiPSC-LESCs are more mature than LECs, but less mature than CECs.

The cornea is in constant contact with air and external environment, and therefore highly exposed to UV-radiation and various pathogens. Consequently, the ocular surface requires protection against the formation of reactive oxygen species (ROS) in the form of proteins with antioxidant properties, as well as proteins involved in immune response. Our data set contains 9 antioxidants, which include peroxiredoxins and superoxide dismutases, and 19 immune response proteins, including interleukin enhancer binding factor 2 (ILF2), IGHA1, IGLC2 and MIF. The ocular surface is known to be a versatile barrier of innate immunity, involving CECs, fibroblasts and Langerhans cells^38^. In this study, protein was extracted from cell pellets, and therefore cytokines and growth factors known to be secreted by the corneal and limbal epithelia, including various interleukins and chemokines such as chemokine (C-C motif) ligand 5 (CCL5), chemokine (C-X-C motif) ligand 2 (CXCL2), or glial cell-derived neurotrophic factor (GDNF)^31^,^38^,^39^, were not detected. In addition, there is evidence suggesting that hPSC-derived cells possess a certain degree of immune privilege^40^,^41^, which would also explain the scarcity of identified immune response proteins. Overall, considering that hPSC-LESCs cultured *in vitro* are unlikely to suffer from exposure to pathogens, as well as UV-radiation and the resulting ROS, it is not surprising that relatively few protective proteins were detected. Another important component of the limbal niche is the blood vessel network of the limbal stroma, which contributes to angiogenesis during wound healing^42^. LESCs have been shown to express both pro- and anti-angiogenic genes, while limbal stromal niche cells possess the ability to differentiate towards angiogenesis progenitors and prevent corneal epithelial differentiation^31^,^42^. Only 10 proteins that may be involved in angiogenesis were identified in our data set, which is not surprising, as the *in vitro* cell culture has no surrounding tissues and blood vessels to interact with, making angiogenesis unnecessary and hence down-regulating the angiogenic markers.

Dyrlund *et al.* have carried out an extensive proteomic analysis of all three layers in the human cornea: they identified 2737 proteins in the corneal epithelium, and quantified either 110 or 663 proteins, depending on the quantification method^43^. Out of the 55 most abundant proteins identified in their study of the corneal epithelium, we detected 44 (Supplementary Table S5). Cytokeratins 3 and 12, as well as MUC16, ALDH3A1, DCN and TGFBI have been previously described to be more prevalent in mature CECs rather than LESCs^31^,^44^,^45^. The results of our study are in line with this: these proteins were detected at higher levels in CECs than in LECs. Moreover, expression profiles of KRT3 and KRT12 suggest that hPSC-LESCs are more mature than LECs, but less mature than CECs. Cytokeratins 5, 7, 8, 14, and 19, HSP70, SERPINA3, and VIM have been detected in primary LESCs in several studies^45^,^46^,^47^. KRT8 is known to be co-localized with KRT15 and VIM, and may be expressed in activated basal cells that are ready to divide and differentiate^48^. HSP70 has been reported to be involved in LEC proliferation, differentiation and migration, co-localizing with p63^47^. In our study, KRT7, KRT8, KRT19 and VIM were expressed at lower levels in CECs and LECs compared to hPSC-LESCs, suggesting that hPSC-LESCs may be more homogeneous cell populations than the native CECs and LECs.

Relative proteomics using the iTRAQ technology is only capable of identifying proteins that are expressed in all analyzed samples. Therefore, iTRAQ methods are known to work best when the differences between samples are minimal, for instance in time-point studies. In this study we compared native tissue from three different human donors with LESC-like cells differentiated from hESCs and hiPSCs. In this case, the differences between the samples are almost certainly high, resulting in a fairly low amount of proteins identified in all samples, thus making the comparison more difficult. Nevertheless, we can clearly see that all samples have similar protein expression profiles, indicating good reliability of the analysis method. Several putative LESC markers, most importantly p63 and ABCG2 could not be identified by means of iTRAQ, meaning that they were absent from at least one of the samples. Most likely, these markers were not present in CECs in detectable amounts, as the central corneal epithelium mainly contains mature cells. Therefore, we verified protein expression of several putative LESC markers that were not identified in the proteomics analysis, namely p63, TCF4, ABCG2 and BMI-1, using flow cytometry and immunofluorescence. Both hESC-LESCs and hiPSC-LESCs were shown to express these proteins, confirming that they do possess LESC-like characteristics.

Overall, it is difficult to draw conclusions regarding the differentiation state of hPSC-LESCs. We postulate that the native human samples collected from the ocular surface were fairly heterogeneous, containing sub-populations of cells in a variety of differentiation states. Furthermore, variation between donors likely affects the outcomes of the study. Significant inter-donor variation was also observed in a microarray study using primary cells collected from the ocular surface even after undergoing expansion *in vitro*^31^. In contrast, several high-throughput microarray studies have shown substantial differences in gene expression between purified populations of CECs and LESCs^6^,^31^,^49^,^50^. Using human cells that have not been expanded *in vitro* has its advantages and disadvantages. On the one hand, they represent the *in vivo* state better, without the risk of proteome alteration caused by prolonged cultivation. On the other hand, it is difficult to obtain a pure population of the desired cell type, especially knowing that only a small percentage of cells in the limbal region are actual stem cells–about 5% of cells in the human limbus are considered LESCs, while the rest are likely TACs at various stages of differentiation^51^. It remains to be seen whether purification of transplantable cell populations via cell sorting becomes a quality requirement for clinical use. In fact, it may be beneficial to include LESCs, TACs and CECs, to better mimic the range of cell types at the ocular surface.

In conclusion, the results of this study show that hPSC-LESCs express at least 485 proteins found in native ocular surface epithelia. Most importantly, proteins involved in cell cycling, proliferation, differentiation and apoptosis, various LESC niche components, and 21 corneal and limbal markers were identified. The overall protein expression profiles of hESC-LESCs and hiPSC-LESCs suggest that the differentiation method is robust and reproducible, yielding cells similar to native ocular surface epithelial cells, with possible clinical efficacy in corneal reconstruction. In the future, more thorough analyses of the hPSC-LESC proteome could be carried out using absolute quantification proteomics. Overall, this is the first study utilizing MS-based proteomics to compare hPSC-LESCs with their *in vivo* counterparts, and an important step towards proteomic characterization of their maturity and clinical applications.

## Methods

### Ethical issues

Human tissue collection was carried out at the University of Debrecen, Hungary. All experimental protocols were approved by the National Medical Ethics Committee of Hungary (14415/2013/EKU—183/2013 and DEOEC RKEB/IKEB 3094/2010), in compliance with the guidelines of the Helsinki Declaration (1964). Hungary follows the EU Member States’ Directive 2004/23/EC on presumed consent practice for tissue collection. All relevant data pertaining to the status of the patient before death were checked before tissue collection.

The use of human embryos for research purposes at the University of Tampere, Finland, was approved by the National Authority for Medicolegal Affairs Finland (Dnro 1426/32/300/05). The institute also has supportive statements of the Ethical Committee of the Pirkanmaa Hospital District to derive, culture, and differentiate hESC lines (Skottman/R05116), and use hiPSC lines derived in other laboratories for ophthalmic research (Skottman/R14023). No new cell lines were derived for this study. All experiments were carried out in accordance with the approved guidelines and regulations.

### Limbal and corneal tissue collection

The workflow of the study is illustrated in Fig. 1. LEC and CEC samples were obtained within 12 hours post-mortem from three donors (males, 70.67 ± 4.16 years of age). CECs were collected by gently scraping the surface of the central cornea, while LECs were collected from the surface of the limbus, the narrow (1–2 mm) zone at the corneoscleral junction. All samples were collected in sterile phosphate buffered saline (PBS), pelleted by centrifugation and stored as dry pellets at −80 °C until protein extraction.

### Cell lines and corneal epithelial differentiation

Two hPSC lines were used in this study. The hESC line (Regea08/017, 46XX) was previously derived at the University of Tampere^52^, while the hiPSC line (UTA.04511.WT 46XY) was reprogrammed from human fibroblasts using the CytoTune®-iPS Sendai Reprogramming Kit (Invitrogen, Carlsbad, CA) by Professor Katriina Aalto-Setälä’s research group at the University of Tampere (Ojala *et al.*, to be published elsewhere). Both cell lines were routinely maintained in hPSC medium consisting of KnockOut Dulbecco’s Modified Eagle’s Medium (KO-DMEM) supplemented with 20% KnockOut Serum Replacement (KO-SR), 2 mM Glutamax, 0.1 mM 2-mercaptoethanol (all from Invitrogen), 1% Non-essential amino acids, 50 U/ml penicillin/streptomycin (both from Lonza Group Ltd, Basel, Switzerland), and 8 ng/ml human basic fibroblast growth factor (bFGF; PeproTech, Rocky Hill, NJ) on mitotically inactivated human foreskin fibroblast (CRL-2429; American Type Culture Collection, ATCC, Manassas, VA) feeder cells. Undifferentiated stem cell colonies were enzymatically passaged onto fresh feeder cell layers at ten-day intervals. To confirm their quality, both cell lines were routinely karyotyped and characterized for their self-renewal and differentiation capacities.

To initiate differentiation towards LESC-like cells, undifferentiated hPSC colonies were manually dissected and cultured as three dimensional cell aggregates for 6–7 days in surface ectoderm induction medium: hPSC medium modified by reducing KO-SR concentration to 15%, increasing bFGF concentration to 50 ng/ml, and adding 10 μM of transforming growth factor β (TGF-β) inhibitor SB-505124 and 10 μM of Wnt inhibitor IWP-2 (both from Sigma-Aldrich, St Louis, MO). After the induction stage, cell aggregates were plated onto 12-well plates (Corning CellBIND; Corning, NY) coated with human placental collagen IV (5 μg/cm^2^; Sigma-Aldrich), and maintained in a defined and serum-free corneal epithelium medium CnT-30 (CELLnTEC Advanced Cell Systems AG, Bern, Switzerland). After a total of 30–35 days in differentiation culture, LESC-like cells were rinsed with PBS twice and treated with TrypLE Select (Invitrogen) for 5 min at +37 °C. Warm cell culture medium was added and single cell suspensions collected to sterile tubes. Cells were pelleted by centrifugation and stored as dry pellets at −80 °C until protein extraction. Three separate biological replicates from each cell line were analyzed.

### Protein extraction

Cell pellets were resuspended in 100–200 μl RIPA lysis buffer, supplemented with 0.1% HALT protein inhibitor cocktail (both from ThermoFisher Scientific, Waltham, MA), and dissociated using a disposable pellet mixer (VWR International, Radnor, PA), followed by mixing with a vortex for 2 min, incubation in an ultrasonic bath for 5 min, and finally on ice for 25 min. Dissociated samples were centrifuged at 14 800 rpm for 15 min at +4 °C, supernatants transferred to clean tubes and their protein concentrations determined using the DC Protein Assay Kit II (Bio-Rad Laboratories, Hercules, CA), according to the manufacturer’s instructions. Bovine serum albumin (BSA; Bio-Rad Laboratories) was used as a standard, and all samples were analyzed in duplicates. Equal amounts of total protein (25 μg/sample) were precipitated by adding six volumes of ice-cold acetone and overnight incubation at −20 °C. The following day, samples were centrifuged at 14 800 rpm for 15 min at +4 °C. The acetone was discarded and samples dried for 10 min, and then subjected to protein digestion.

### Protein digestion

Dried protein pellets were resuspended in dissolution buffer (50 mM ABC solution; Sigma-Aldrich), supplemented with 2% sodium dodecyl sulfate (SDS; Fluka, Sigma-Aldrich), mixed with a vortex for 2 min and centrifuged at 14 000 rpm for 1 min. Disulfide bonds were reduced in 50 mM Tris-(2-carboxyethyl)-phosphine (TCEP; Fluka, Sigma-Aldrich) for 1 h at +60 °C with interval mixing (alternating 15 min static and 1 min at 1250 rpm), followed by centrifugation at 14 000 rpm for 1 min. Samples were then transferred to 30 kDa size exclusion filters (Pall Corporation, Port Washington, NY) and washed twice with 75% urea solution, with centrifugation at 12 100 rpm for 15 min after each wash. Cysteine residues were blocked with iodoacetamide solution (5 mM; Sigma-Aldrich) for 20 min, protected from light. The samples were then washed four times with urea solution, and three times with ABC solution, with centrifugation at 12 100 rpm for 10 min after each wash. Trypsin (AB Sciex, Concord, Canada) diluted in ABC solution was added (1 μg/sample), followed by incubation at +37 °C for 16 h with interval mixing (alternating 5 min static and 15 min at 1250 rpm). Trypsinized samples were cooled to +4 °C, centrifuged at 14 000 rpm for 1 min, and washed with ABC solution twice, with centrifugation at 14 000 rpm for 10 min after each wash. Then, NaCl solution was added, samples centrifuged at 14 000 rpm for 15 min, and dried by vacuum centrifugation for up to 1 h. Finally, sample desalting was done using Pierce C18 tips (Thermo Fisher Scientific) as follows: tips were first rinsed twice with 50% acetonitrile (ACN; Fluka, Sigma-Aldrich) and twice with 0.1% trifluoroacetic acid (TFA; Sigma-Aldrich) to balance the C18 phase. Samples were dissolved in 0.1% TFA and flushed in C18 tips 5–10 times, then washed twice with 2.5% ACN in 0.1% TFA, and slowly aspirated into clean tubes with 80% ACN in 0.1% formic acid (FA; Fluka, Sigma-Aldrich). The desalted samples were dried by vacuum centrifugation for up to 1 h.

### iTRAQ labeling

Each iTRAQ reagent (114, 115, 116 and 117 isobaric tags) was reconstituted in ethanol, and peptide samples were reconstituted in iTRAQ dissolution buffer (AB Sciex). Samples in sets of three biological replicates were labeled with the iTRAQ reagents as follows: hESC-LESC with 114 isobaric tag, hiPSC-LESC with 115 isobaric tag, human CEC with 116 isobaric tag, and human LEC with 117 isobaric tag. The labeled samples were incubated for 2 h at room temperature with interval mixing (alternating 1 min static and 15 min at 1200 rpm), centrifuged at 13 500 rpm for 5 min, and pooled together into three groups each containing one biological replicate of the studied samples. These groups were handled and analyzed separately. The pooled samples were dried by vacuum centrifugation. Sample clean-up and desalting was carried out using Macro Spin Column Filters (Nest GroupInc, Southborough, MA), filtering and washing samples several times with ACN and triethylammonium bicarbonate (TEAB; Fluka, Sigma-Aldrich) buffers. The samples were dried by vacuum centrifugation for about 1 h, and stored at −20 °C until analysis.

### Mass spectrometry analysis

The three groups of labeled samples, each containing one biological replicate of the studied samples, were analyzed in duplicate by Nano-RPLC-TripleTOF instrumentation using Eksigent 425 NanoLC coupled to high speed TripleTOF™ mass spectrometer (AB Sciex). A capillary RP-LC column (cHiPLC^®^ ChromXP C18-CL, 3 μm particle size, 120 Å, 75 μm i.d × 15 cm; Eksigent, Concord, Canada) was used for LC separation of peptides. Samples were first loaded into trap column (cHiPLC^®^ ChromXP C18-CL, 3 μm particle size, 120 Å, 75 μm i.d × 5 mm) from autosampler and flushed for 10 min at a flow rate of 2 μl/min (in 2% ACN, 0.1% FA). The system was then switched to the line with analytical column. Labeled samples were analyzed with 120 min six-step gradient at a flow rate of 300 nl/min, using eluent A (0.1% FA in 1% ACN) and eluent B (0.1% FA in ACN), changing the eluent B concentration as follows: 5–7% over 2 min, 7–24% over 55 min, 24–40% over 29 min, 40–60% over 6 min, 60–90% over 2 min, kept at 90% for 15 min, decreased down to 5% over 0.1 min, and kept at 5% for 13 min.

The following key parameters for TripleTOF mass spectrometer were used: ion spray voltage floating (ISVF) 2300 V, curtain gas (CUR) 30, interface heater temperature (IHT) 125 °C, ion source gas 1 (GS1) 13, declustering potential (DP) 80 V. All data were acquired with information-dependent acquisition (IDA) mode with Analyst TF 1.5 software (AB Sciex). For IDA parameters, 0.25 s MS survey scan in the mass range of 350–1250 mass/charge (m/z) were followed by 30 MS/MS scans in the mass range of 100–1500 Da (total cycle time 2.051 s). Switching criteria were set to ions greater than 350 m/z and smaller than 1250 m/z, with charge state 2–5 and an abundance threshold of more than 120 counts. Former target ions were excluded for 8 s. IDA rolling collision energy parameters script was used for automatically controlling collision energy.

### Data processing and analysis

ProteinPilot software version 4.0.8085 (AB Sciex) was used to analyze MS/MS data and searched against the International Protein Index (IPI) protein database version 3.87 for protein identification. Some important settings in the Paragon search algorithm in ProteinPilot were configured as follows. Sample type: identification, Cys-alkylation: MMTS, Digestion: Trypsin, Instrument: TripleTof 5600+, Search effort: thorough ID. False discovery rate (FDR) analysis was performed in ProteinPilot, and FDR < 1% was set for protein identification. To make use of the most suitable proteomic tools, all identified proteins were converted to the Universal Protein Knowledgebase (UniProtKB) accession numbers using the commonly-available cross-reference systems CRONOS and PICR^53^,^54^. Log transformation was applied to the data in order to reduce the effects of extreme values and to allow the data set to follow a log normal distribution. Central tendency normalization was implemented to reduce the effects of systematic bias. For a more detailed analysis, only proteins that were detected in at least two biological replicates were used. Biological interpretation of the results was carried out with the help of PANTHER (Protein Analysis Through Evolutionary Relationships) Classification System, and DAVID (Database for Annotation, Visualization and Integrated Discovery) Functional Annotation Database^55^,^56^. Mean fold changes and standard deviations were calculated from biological and technical replicates for each sample. Pie charts and scatter plots were created and modified in Microsoft Excel, based on PANTHER and DAVID protein classification and clustering.

### Flow cytometry

Protein expression of the putative LESC marker BMI-1 in hESC-LESCs and hiPSC-LESCs was verified using flow cytometry. Cells were detached using TrypLE Select, and single cell suspensions collected to sterile tubes through 40 μm cell strainers. Prior to staining, cells were washed twice with 0.5% BSA and 0.01% NaN_3_ (Sigma-Aldrich), fixed with 4% paraformaldehyde (PFA) for 10 min, then washed twice with BD Perm/Wash™ Buffer (BD Biosciences, Franklin Lakes, NJ) and incubated in the same buffer for 15 min. Cells were then incubated for 30 min on ice, protected from light, with FITC-conjugated mouse anti-human BMI-1 antibody or FITC-conjugated mouse anti-human IgG2A isotype control (both from R&D Systems, Minneapolis, MN). After two final washes with BD Perm/Wash™ Buffer, cells were analyzed using BD Accuri C6 Flow Cytometer (BD Biosciences).

### Immunofluorescence

Protein expression of putative LESC markers ΔNp63, TCF4 and ATP-binding cassette sub-family G member 2 (ABCG2) in hESC-LESCs and hiPSC-LESCs was verified with immunofluorescence stainings, as previously described^18^. The following primary antibodies were used: goat anti-p63 (Santa Cruz Biotechnology, sc-25039, diluted 1:100), mouse anti-TCF4 (Santa Cruz Biotechnology, sc-166699, diluted 1:400), and mouse anti-ABCG2 (Merck Millipore, MAB4155, diluted 1:200). Their detection was carried out using the following secondary antibodies: Alexa 568-conjugated donkey anti-goat (Molecular Probes, A-11057, diluted 1:800) and Alexa 488-conjugated donkey anti-mouse (Molecular Probes, A-21202, diluted 1:800). Mounting medium containing DAPI (VectaShield, Vector Laboratories Inc., Burlingame, CA) was used for visualization of nuclei. Images were captured using Zeiss LSM 700 confocal microscope (Carl Zeiss, Jena, Germany).

## Additional Information

**How to cite this article**: Mikhailova, A. *et al.* Comparative proteomics reveals human pluripotent stem cell-derived limbal epithelial stem cells are similar to native ocular surface epithelial cells. *Sci. Rep.* **5**, 14684; doi: 10.1038/srep14684 (2015).

## Supplementary Material

Supplementary Table S1

Supplementary Tables

## Figures and Tables

**Table 1:** Cell proliferation proteins.

**Protein**	**CEC:hESC-LESC**	**CEC:hiPSC-LESC**	**LEC:hESC-LESC**	**LEC:hiPSC-LESC**
Positive regulation of cell proliferation				
CALR	−1.4 (±0.2)	1.2 (±1.2)	−1.1 (±0.3)	1.3 (±0.9)
CAPN1	1.9 (±0.6)	1.3 (±0.3)	1.9 (±0.3)	1.3 (±0.3)
CAPNS1	2.5 (±1.0)	2.5 (±1.4)	2.4 (±1.1)	2.4 (±1.2)
CDH13*	1.5 (±0.4)	11.3 (±15.1)	1.9 (±0.7)	12.7 (±16.9)
CLU	3.6 (±1.3)	3.7 (±0.9)	2.7 (±1.0)	2.8 (±0.8)
COL18A1*	5.4 (±3.8)	2.1 (±0.3)	7.4 (±7.2)	2.9 (±1.5)
EIF5A	−2.8 (±0.2)	−2.0 (±0.2)	−3.3 (±0.1)	−2.5 (±0.04)
GNAI2	1.1 (±0.2)	1.1 (±0.2)	−1.0 (±0.3)	−1.3 (±0.3)
ITGB1	1.1 (±0.4)	1.3 (±0.7)	−1.1 (±0.3)	1.1 (±0.4)
NME2*	−2.2 (±0.1)	−2.3 (±0.2)	−2.3 (±0.1)	−2.7 (±0.1)
NPM1*	−6.4 (±0.03)	−4.9 (±0.1)	−3.1 (±0.2)	−3.1 (±0.1)
PRDX3	−1.5 (±0.1)	1.2 (±0.4)	−1.1 (±0.2)	1.6 (±0.8)
RPS15A	−3.7 (±0.04)	−1.2 (±0.4)	−3.1 (±0.02)	1.1 (±0.6)
RPS4X	−3.5 (±0.1)	−3.3 (±0.1)	−2.7 (±0.2)	−2.9 (±0.1)
RPS9	−1.7 (±0.3)	−2.3 (N/A)	−1.4 (±0.5)	−2.7 (N/A)
S100A6	2.1 (±0.9)	2.3 (±1.1)	1.8 (±0.6)	1.9 (±0.8)
SSR1	2.8 (±0.9)	2.9 (±1.9)	2.6 (±1.6)	2.2 (±1.0)
TGM2	−1.0 (±0.3)	−1.8 (±0.1)	1.9 (±0.6)	1.1 (±0.2)
Negative regulation of cell proliferation				
ASPH	2.3 (±0.4)	2.5 (±0.4)	1.8 (±0.3)	2.0 (±0.4)
ATP5A1	1.7 (±0.3)	1.6 (±0.5)	1.6 (±0.4)	1.6 (±0.4)
CAV1	−1.6 (±0.2)	−2.8 (N/A)	−1.1 (±0.1)	−2.7 (N/A)
CDH13*	1.5 (±0.4)	11.3 (±15.1)	1.9 (±0.7)	12.7 (±16.9)
COL18A1*	5.4 (±3.8)	2.1 (±0.3)	7.4 (±7.2)	2.9 (±1.5)
COMT	1.3 (±0.7)	2.2 (±1.4)	1.6 (±0.8)	2.6 (±1.5)
FTH1	1.5 (±0.5)	1.7 (±0.5)	1.0 (±0.4)	1.3 (±0.9)
GPNMB	1.1 (±0.5)	1.1 (±0.7)	2.7 (±0.8)	3.2 (±2.9)
KRT4	1.9 (±1.5)	1.6 (±0.4)	1.5 (±0.9)	1.3 (±0.1)
KRT5	2.5 (±2.4)	2.1 (±1.9)	−1.9 (±0.3)	−2.4 (±0.3)
NME2*	−2.2 (±0.1)	−2.3 (±0.2)	−2.3 (±0.1)	−2.7 (±0.1)
NPM1*	−6.4 (±0.03)	−4.9 (±0.1)	−3.1 (±0.2)	−3.1 (±0.1)
PHB	−1.4 (±0.2)	−1.3 (±0.2)	−1.1 (±0.2)	−1.0 (±0.3)
PTGES	4.3 (±1.5)	1.5 (±0.8)	3.3 (±0.8)	1.1 (±0.5)
S100A11	1.2 (±0.5)	1.3 (±1.6)	1.6 (±0.7)	1.6 (±1.4)
SFN	−1.1 (±0.2)	−1.2 (±0.2)	−1.0 (±0.4)	−1.1 (±0.2)
SOD2	−1.2 (±0.2)	1.1 (±0.4)	2.0 (±1.3)	2.3 (±0.9)

Expression differences in human native CECs and LECs compared to hESC-LESCs and hiPSC-LESCs, presented as mean fold changes ± standard deviation. Asterisks (*) denote proteins that participate both in positive and negative regulation of cell proliferation.
