# Supplementary material for: Comparative proteomics reveals human pluripotent stem cell-derived limbal epithelial stem cells are similar to native ocular surface epithelial cells
Source: Sci Rep. 2015 Oct 1;5:14684. doi: 10.1038/srep14684 (PMC4589773; doi:10.1038/srep14684)
Supplement: Supplementary Tables [file srep14684-s2.pdf]

Comparative proteomics reveals human pluripotent stem cell-derived limbal epithelial stem cells are similar to native ocular surface epithelial cells

Alexandra Mikhailova, Antti Jylhä, Jochen Rieck, Janika Nättinen, Tanja Ilmarinen, Zoltán Veréb, Ulla Aapola, Roger Beuerman, Goran Petrovski, Hannu Uusitalo, Heli Skottman

Supplementary Table S2. Proteins involved in cell cycling, differentiation and apoptosis. Expression differences in primary CECs and LECs compared to hESC-LESCs and hiPSC-LESCs, presented as mean fold changes and standard deviation. Related to Figure 4.

UniProt	Protein symbol	Full name	CEC : hESC-LESCs		LEC : hESC-LESCs		CEC : hiPSC-LESCs		LEC : hiPSC-LESCs	
Cell cycling			Mean	StDev	Mean	StDev	Mean	StDev	Mean	StDev
P04083	ANXA1	Annexin A1	-1.559	0.099	1.180	0.330	-2.718	0.044	-1.406	0.294
P27797	CALR	Calreticulin	-1.426	0.233	-1.104	0.297	1.183	1.219	1.343	0.958
B4DGU4	CTNNB1	Beta-catenin	-1.041	0.817	1.269	0.943	1.194	0.669	1.641	0.860
Q13561	DCTN2	Dynactin subunit 2	1.537	1.392	1.517	1.077	2.013	2.155	1.874	1.576
P54652	HSPA2	Heat shock-related 70 kDa protein 2	1.427	0.201	1.123	0.102	-1.742	0.010	-2.268	0.032
Q12906	ILF3	Interleukin enhancer binding factor 3	-1.405	0.314	-1.598	0.288	-1.429	0.199	-1.687	0.199
P05556	ITGB1	Integrin beta-1	1.093	0.395	-1.091	0.307	1.316	0.656	1.064	0.401
Q07666	KHDRBS1	KH domain-containing, RNA-binding, signal transduction-associated protein 1	-2.051	0.048	-1.694	0.315	-3.064	0.022	-2.655	0.133
P08729	KRT7	Keratin, type II cytoskeletal 7	-7.534	0.096	-7.395	0.084	-14.231	0.056	-17.539	0.027
P35579	MYH9	Myosin, heavy chain 9, non-muscle	-4.227	0.046	-4.136	0.063	-3.408	0.121	-3.455	0.133
P06748	NPM1	Nucleophosmin	-6.375	0.026	-3.132	0.161	-4.881	0.102	-3.130	0.061
Q8WUM4	PDCD6IP	Programmed cell death 6-interacting protein	1.344	0.237	1.405	0.062	1.669	0.970	1.662	0.581
Q53YE8	PSMA1	Proteasome subunit alpha type 1	-1.808	0.058	-1.297	0.129	-1.422	0.492	1.014	0.870
P25787	PSMA2	Proteasome subunit alpha type 2	1.488	0.688	1.692	0.679	1.136	0.476	1.271	0.395
P25788	PSMA3	Proteasome subunit alpha type 3	-1.202	0.366	1.050	0.595	1.231	0.552	1.688	1.216
H0YN18	PSMA4	Proteasome subunit alpha type 4	1.520	0.385	1.534	1.074	-1.546	0.074	-1.700	0.259
P60900	PSMA6	Proteasome subunit alpha type 6	-1.547	0.042	-1.247	0.094	-1.115	0.261	1.040	0.438
O14818	PSMA7	Proteasome subunit alpha type 7	-1.636	0.147	-1.372	0.363	-1.210	0.321	1.010	0.347
P20618	PSMB1	Proteasome subunit beta type 1	-1.089	0.187	-1.165	0.108	-1.029	0.799	-1.179	0.542
O00232	PSMD12	26S proteasome non-ATPase regulatory subunit 12	-1.872	0.061	-2.153	0.066	1.034	0.578	-1.095	0.577
O43242	PSMD3	26S proteasome non-ATPase regulatory subunit 3	-4.264	0.051	-2.863	0.126	-1.751	0.579	-1.542	0.423
P51665	PSMD7	26S proteasome non-ATPase regulatory subunit 7	1.993	1.189	2.005	1.397	-1.034	0.101	-1.097	0.024
Q06323	PSME1	Proteasome activator complex subunit1	-1.058	0.388	1.403	0.240	-1.403	0.605	-1.002	0.726
B5MDF5	RAN	GTP-binding nuclear protein Ran	-4.250	0.079	-4.799	0.039	-2.188	0.313	-2.728	0.147
P62979	RPS27A	Ubiquitin-40S ribosomal protein S27a	1.338	0.455	1.380	0.710	2.278	1.606	2.169	1.302
P06703	S100A6	Protein S100-A6	2.144	0.955	1.779	0.584	2.336	1.104	1.899	0.759
B5MCX3	SEPT2	Septin 2	-2.207	0.008	-1.621	0.234	-2.914	0.011	-2.176	0.192
Q5JP53	TUBB	Tubulin, beta	-6.727	0.033	-7.560	0.028	-6.062	0.033	-6.949	0.035
Cell Differentiation			Mean	StDev	Mean	StDev	Mean	StDev	Mean	StDev
P61158	ACTR3	Actin-related protein 3	1.499	0.627	2.247	1.248	-1.283	0.256	1.088	0.479

Q8IVF2	AHNAK2	Protein AHNAK2	1.431	0.586	1.200	0.546	2.220	1.107	1.935	1.298
P04083	ANXA1	Annexin A1	-1.559	0.099	1.180	0.330	-2.718	0.044	-1.406	0.294
P27797	CALR	Calreticulin	-1.426	0.233	-1.104	0.297	1.183	1.219	1.343	0.958
P20810	CAST	Calpastatin	1.943	0.822	1.485	0.777	1.806	0.926	1.733	0.909
Q59E85	CAV1	Caveolin 1	-1.607	0.215	-1.113	0.086	-2.818	N/A	-2.671	N/A
B4E1U9	CDC42	Cell division control protein 42	1.211	0.197	1.186	0.148	1.182	0.279	1.156	0.305
P12830	CDH1	Cadherin 1, E-cadherin (epithelial)	1.782	0.545	1.903	0.431	1.697	0.447	1.760	0.188
P23528	CFL1	Cofilin-1	-2.636	0.042	-2.305	0.154	-2.903	0.056	-2.522	0.189
P10909	CLU	Clusterin	3.584	1.284	2.726	1.009	3.652	0.970	2.796	0.837
P39060	COL18A1	Collagen alpha-1 (XVIII) chain	5.397	3.806	7.418	7.192	2.107	0.310	2.916	1.534
B4DGU4	CTNNB1	Beta-catenin	-1.041	0.817	1.269	0.943	1.194	0.669	1.641	0.860
P15924	DSP	Desmoplakin	1.425	0.786	-1.080	0.600	-1.411	0.112	-3.181	0.018
Q96FJ2	DYNLL2	Dynein light chain 2, cytoplasmic	1.929	0.570	1.345	0.318	5.951	3.873	3.924	2.807
O75369	FLNB	Filamin-B	-6.400	0.053	-6.778	0.052	-6.621	0.031	-7.222	0.032
Q14956	GPNMB	Transmembrane glycoprotein NMB	1.086	0.477	2.759	0.782	1.122	0.718	3.195	2.869
P06396	GSN	Gelsolin	2.291	0.613	2.078	0.152	2.227	0.460	2.000	0.325
P62805	HIST1H4A	Histone H4	1.213	0.482	1.511	0.380	1.440	0.177	1.809	0.215
Q99729	HNRNPAB	Heterogeneous nuclear ribonucleoprotein AB	-1.328	0.050	-1.161	0.026	-1.121	0.116	-1.204	0.181
Q99714	HSD17B10	3-hydroxyacyl-CoA dehydrogenase type-2	1.085	0.392	-1.287	0.320	1.282	1.078	-1.158	0.635
P54652	HSPA2	Heat shock-related 70 kDa protein 2	1.427	0.201	1.123	0.102	-1.742	0.010	-2.268	0.032
P23229	ITGA6	Integrin alpha-6	1.153	0.404	1.049	0.137	-1.456	0.489	-1.742	0.325
P05556	ITGB1	Integrin beta-1	1.093	0.395	-1.091	0.307	1.316	0.656	1.064	0.401
P02533	KRT14	Keratin, type I cytoskeletal 14	1.163	0.267	-2.056	0.296	-1.186	0.217	-2.901	0.219
P08272	KRT19	Keratin, type I cytoskeletal 19	-6.246	0.082	-4.112	0.112	-14.013	0.056	-10.301	0.071
P12035	KRT3	Keratin, type II cytoskeletal 3	6.121	2.465	-1.701	0.337	10.922	2.590	-1.424	0.401
P19013	KRT4	Keratin, type II cytoskeletal 4	1.870	1.504	1.519	0.973	1.581	0.439	1.336	0.072
P13647	KRT5	Keratin, type II cytoskeletal 5	2.536	2.378	-1.852	0.290	2.068	1.917	-2.448	0.257
P09382	LGALS1	Galectin-1	-9.037	0.029	-9.467	0.068	-4.176	0.260	-7.875	0.063
P17931	LGALS3	Galectin-3	1.800	1.118	1.366	0.481	1.658	0.914	1.279	0.448
P35579	MYH9	Myosin, heavy chain 9, non-muscle	-4.227	0.046	-4.136	0.063	-3.408	0.121	-3.455	0.133
P22392	NME2	Nucleoside diphosphate kinase B	-2.227	0.148	-2.331	0.139	-2.347	0.175	-2.704	0.083
Q9Y639	NPTN	Neuroplastin	1.547	0.855	1.332	0.757	2.456	2.240	1.935	1.576
P30086	PEBP1	Phosphatidylethanolamine-binding protein 1	-1.394	0.126	-1.238	0.388	-1.309	0.219	-1.106	0.563
O60437	PPL	Periplakin	2.004	0.466	1.633	0.227	1.543	0.607	1.191	0.269
P30153	PPP2R1A	Serine/threonine-protein phosphatase 2A 65 kDa regulatory subunit A	1.046	0.776	-1.093	0.333	-1.022	1.166	-1.357	0.557
P32119	PRDX2	Peroxiredoxin-2	-1.483	0.168	-1.277	0.357	-1.120	0.422	1.063	0.748
P30048	PRDX3	Thioredoxin-dependent peroxide reductase, mitochondrial	-1.519	0.109	-1.145	0.234	1.187	0.377	1.608	0.785
P63000	RAC1	Ras-related C3 botulinum toxin substrate 1	-1.438	0.190	1.054	0.383	-1.858	0.138	-1.116	0.044
B5MDF5	RAN	GTP-binding nuclear protein Ran	-4.250	0.079	-4.799	0.039	-2.188	0.313	-2.728	0.147
P35268	RPL22	60S ribosomal protein L22	-1.004	0.802	1.338	1.406	-1.589	0.134	-1.580	0.091
P39019	RPS19	40S ribosomal protein S19	1.027	0.381	-1.122	0.446	1.135	0.343	-1.042	0.345
P62979	RPS27A	Ubiquitin-40S ribosomal protein S27a	1.338	0.455	1.380	0.710	2.278	1.606	2.169	1.302
Q9NQC3	RTN4	Reticulon-4	1.424	0.940	1.202	0.762	1.664	1.132	1.379	0.908
P26447	S100A4	Protein S100-A4	7.593	3.718	6.884	1.880	9.307	3.698	8.669	1.764
P06703	S100A6	Protein S100-A6	2.144	0.955	1.779	0.584	2.336	1.104	1.899	0.759
O60613	SEP15	15 kDa selenoprotein	4.500	3.579	5.490	1.893	5.154	4.948	5.312	0.463
B5MCX3	SEPT2	Septin 2	-2.207	0.008	-1.621	0.234	-2.914	0.011	-2.176	0.192
P31947	SFN	Stratifin, 14-3-3 protein sigma	-1.144	0.227	-1.031	0.398	-1.175	0.169	-1.126	0.172
P00441	SOD1	Superoxide dismutase [Cu-Zn]	1.080	0.570	1.253	0.782	1.126	0.335	1.270	0.518
P04179	SOD2	Superoxide dismutase 2	-1.203	0.236	1.959	1.288	1.135	0.409	2.303	0.948
P55327	TPD52	Tumor protein D52	2.047	0.685	1.927	0.760	3.060	1.859	2.939	2.419
P09493	TPM1	Tropomyosin alpha-1 chain	-5.174	0.065	-3.729	0.142	-9.668	0.015	-7.830	0.030

Q5JP53	TUBB	Tubulin, beta	-6.727	0.033	-7.560	0.028	-6.062	0.033	-6.949	0.035
B4E1P0	UPK1B	Uroplakin-1b	2.893	2.557	1.013	0.935	3.110	0.832	1.087	0.088
P13010	XRCC5	X-ray repair cross-complementing protein 5	-1.004	0.322	1.018	0.112	1.543	1.567	1.635	1.768
P12956	XRCC6	X-ray repair cross-complementing protein 6	1.011	0.389	-1.008	0.478	1.547	1.230	1.404	1.103
P62258	YWHAE	14-3-3 protein epsilon	-1.181	0.266	-1.223	0.156	1.226	0.499	1.079	0.401
P61981	YWHAG	14-3-3 protein gamma	1.105	0.720	-1.050	0.595	-1.045	0.639	-1.247	0.437
		Apoptosis	**Mean**	**StDev**	**Mean**	**StDev**	**Mean**	**StDev**	**Mean**	**StDev**
O43707	ACTN4	Alpha-actinin-4	-1.496	0.170	-1.483	0.221	-1.778	0.165	-1.808	0.155
P02768	ALB	Serum albumin	-1.559	0.099	1.180	0.330	-2.718	0.044	-1.406	0.294
P04083	ANXA1	Annexin A1	1.064	0.408	1.079	0.382	1.316	0.629	1.256	0.495
Q6P452	ANXA4	Annexin A4	-3.682	0.063	-2.609	0.050	-4.851	0.066	-3.570	0.055
H2RAT1	ANXA5	Annexin A5	2.280	0.489	1.957	0.476	2.392	0.816	2.031	0.677
P51572	BCAP31	B-cell receptor-associated protein 31	4.476	2.327	2.598	1.286	5.991	3.777	3.394	2.104
Q9BXK5	BCL2L13	Bcl-2-like protein 13	-1.426	0.233	-1.104	0.297	1.183	1.219	1.343	0.958
P27797	CALR	Calreticulin	1.782	0.545	1.903	0.431	1.697	0.447	1.760	0.188
P12830	CDH1	Cadherin 1, E-cadherin (epithelial)	1.494	0.429	1.845	0.676	11.295	15.135	12.714	16.914
P55290	CDH13	Cadherin-13	-2.636	0.042	-2.305	0.154	-2.903	0.056	-2.522	0.189
P23528	CFL1	Cofilin-1	3.584	1.284	2.726	1.009	3.652	0.970	2.796	0.837
P10909	CLU	Clusterin	5.397	3.806	7.418	7.192	2.107	0.310	2.916	1.534
P39060	COL18A1	Collagen alpha-1(XVIII) chain	-1.041	0.817	1.269	0.943	1.194	0.669	1.641	0.860
B4DGU4	CTNNB1	Beta-catenin	1.217	0.155	1.174	0.245	1.265	0.287	1.163	0.243
B4DL49	CTSB	Cathepsin B	2.032	1.354	1.633	0.767	1.638	0.704	1.374	0.549
C9JFR7	CYCS	Cytochrome C	1.066	0.712	1.112	0.484	-1.106	0.558	1.106	0.733
P61803	DAD1	Dolichyl-diphosphooligosaccharide--protein glycosyltransferase subunit DAD1	1.330	0.539	1.356	0.806	1.154	0.252	1.122	0.284
Q9NR28	DIABLO	Diablo homolog, mitochondrial precursor	-2.829	0.166	-3.317	0.056	-2.013	0.236	-2.463	0.040
P63241	EIF5A	Eukaryotic translation initiation factor 5A-1	2.291	0.613	2.078	0.152	2.227	0.460	2.000	0.325
P06396	GSN	Gelsolin	1.575	0.556	1.771	0.938	1.844	0.621	2.054	1.123
P09211	GSTP1	Glutathione S-transferase P	-1.687	0.285	-2.017	0.134	-1.499	0.279	-1.814	0.115
P09429	HMGB1	High mobility group protein B1	-1.168	0.214	-1.247	0.251	1.180	0.283	1.080	0.333
P14625	HSP90B1	Endoplasmin	2.235	0.640	2.467	0.287	2.095	0.606	2.248	0.268
P08107	HSPA1A/1B	Heat shock 70 kDa protein 1A/1B	-1.741	0.146	-1.664	0.200	-1.431	0.108	-1.421	0.166
P11021	HSPA5	78 kDa glucose-regulated protein	-1.066	0.269	-1.514	0.143	1.293	0.438	-1.144	0.121
P38646	HSPA9	Stress-70 protein, mitochondrial	3.420	1.132	3.861	0.577	2.670	1.182	2.994	0.467
P04792	HSPB1	Heat shock protein beta-1	-1.530	0.061	-1.463	0.101	-1.332	0.145	-1.266	0.286
P10809	HSPD1	60 kDa heat shock protein, mitochondrial	1.206	0.231	1.276	0.412	1.782	0.580	1.828	0.521
P61604	HSPE1	10 kDa heat shock protein, mitochondrial	-9.037	0.029	-9.467	0.068	-4.176	0.260	-7.875	0.063
P09382	LGALS1	Galectin-1	2.299	0.942	2.449	1.341	4.123	3.541	4.326	4.481
P47929	LGALS7	Galectin-7	1.669	0.390	1.910	0.576	2.550	1.694	3.175	2.660
P14174	MIF	Macrophage migration inhibitory factor	1.895	2.263	1.597	1.168	1.419	1.034	1.282	0.294
Q9P0J0	NDUFA13	NADH dehydrogenase [ubiquinone] 1 alpha subcomplex subunit 13	2.367	2.455	2.110	2.334	1.119	0.465	1.203	0.599
P28331	NDUFS1	NADH-ubiquinone oxidoreductase 75 kDa subunit, mitochondrial	-2.227	0.148	-2.331	0.139	-2.347	0.175	-2.704	0.083
P22392	NME2	Nucleoside diphosphate kinase B	-6.375	0.026	-3.132	0.161	-4.881	0.102	-3.130	0.061
P06748	NPM1	Nucleophosmin	2.981	0.702	1.903	0.890	3.261	0.896	1.914	0.686
B4DLR8	NQO1	NAD(P)H dehydrogenase, quinone 1	1.308	0.394	1.077	0.457	2.080	1.629	1.518	0.932
C9JND6	PDCD10	Programmed cell death protein 10	1.344	0.237	1.405	0.062	1.669	0.970	1.662	0.581
Q8WUM4	PDCD6IP	Programmed cell death 6-interacting protein	-1.019	0.118	-1.017	0.171	-1.058	0.058	-1.065	0.079
P30101	PDIA3	Protein disulfide-isomerase A3	-1.396	0.166	-1.068	0.248	-1.313	0.208	-1.021	0.293
P35232	PHB	Prohibitin	1.046	0.776	-1.093	0.333	-1.022	1.166	-1.357	0.557

P30153	PPP2R1A	Serine/threonine-protein phosphatase 2A 65 kDa regulatory subunit A	-1.037	0.146	-1.108	0.173	1.177	0.218	1.077	0.149
Q06830	PRDX1	Peroxiredoxin-1	-1.483	0.168	-1.277	0.357	-1.120	0.422	1.063	0.748
P32119	PRDX2	Peroxiredoxin-2	-1.519	0.109	-1.145	0.234	1.187	0.377	1.608	0.785
P30048	PRDX3	Thioredoxin-dependent peroxide reductase, mitochondrial	2.405	0.213	2.286	0.306	1.854	0.732	1.719	0.561
P30044	PRDX5	Peroxiredoxin-5, mitochondrial	-1.438	0.190	1.054	0.383	-1.858	0.138	-1.116	0.044
P63000	RAC1	Ras-related C3 botulinum toxin substrate 1	1.338	0.455	1.380	0.710	2.278	1.606	2.169	1.302
P62979	RPS27A	Ubiquitin-40S ribosomal protein S27a	-2.059	0.253	-1.991	0.231	-1.385	0.295	-1.209	0.408
P23396	RPS3	40S ribosomal protein S3	1.170	0.280	1.056	0.264	2.809	1.725	2.374	1.376
O95197	RTN3	Reticulon-3	1.424	0.940	1.202	0.762	1.664	1.132	1.379	0.908
Q9NQC3	RTN4	Reticulon-4	-1.144	0.227	-1.031	0.398	-1.175	0.169	-1.126	0.172
P31947	SFN	Stratifin 14-3-3 protein sigma	1.080	0.570	1.253	0.782	1.126	0.335	1.270	0.518
P00441	SOD1	Superoxide dismutase [Cu-Zn]	-1.203	0.236	1.959	1.288	1.135	0.409	2.303	0.948
P04179	SOD2	Superoxide dismutase 2	-1.026	0.262	1.881	0.570	-1.837	0.117	1.057	0.200
P21980	TGM2	Protein-glutamine gamma-glutamyltransferase 2	2.118	1.347	1.573	0.539	4.856	5.949	2.991	2.240
Q9H3N1	TMX1	Thioredoxin-related transmembrane protein 1	-6.727	0.033	-7.560	0.028	-6.062	0.033	-6.949	0.035
Q5JP53	TUBB	Tubulin, beta	-1.776	0.162	-1.532	0.199	-1.615	0.212	-1.405	0.306
P68371	TUBB2C	Tubulin beta-2C chain	-1.155	0.193	-1.150	0.230	-1.013	0.402	-1.055	0.413
B2RDM2	TXNDC5	Thioredoxin domain-containing protein 5	-2.114	0.062	-1.872	0.193	-1.905	0.140	-1.749	0.264
P55072	VCP	Transitional endoplasmic reticulum ATPase	1.753	0.363	1.539	0.511	1.744	0.454	1.480	0.327
P21796	VDAC1	Voltage-dependent anion-selective channel protein 1	-1.004	0.322	1.018	0.112	1.543	1.567	1.635	1.768
P13010	XRCC5	X-ray repair cross-complementing protein 5	1.109	0.091	1.034	0.544	1.106	0.564	-1.020	0.509
P31946	YWHAB	14-3-3 protein beta/alpha	-1.181	0.266	-1.223	0.156	1.226	0.499	1.079	0.401
P62258	YWHAE	14-3-3 protein epsilon	-1.046	0.181	-1.016	0.164	-1.234	0.204	-1.235	0.089
P63104	YWHAZ	14-3-3 protein zeta/delta	-1.496	0.170	-1.483	0.221	-1.778	0.165	-1.808	0.155

Supplementary Table S3. Niche components of the ocular surface. Expression differences in primary CECs and LECs compared to hESC-LESCs and hiPSC-LESCs, presented as mean fold changes and standard deviation. Related to Figure 5.

UniProt	Protein symbol	Full name	CEC : hESC-LESCs		LEC : hESC-LESCs		CEC : hiPSC-LESCs		LEC : hiPSC-LESCs	
Cell adhesion			Mean	StDev	Mean	StDev	Mean	StDev	Mean	StDev
P12830	CDH1	Cadherin 1, E-cadherin (epithelial)	1.782	0.545	1.903	0.431	1.697	0.447	1.760	0.188
P55290	CDH13	Cadherin 13	1.494	0.429	1.845	0.676	11.295	15.135	12.714	16.914
Q14002	CEACAM7	Carcinoembryonic antigen-related cell adhesion molecule 7	-1.473	0.215	4.262	3.094	-1.787	0.316	3.052	1.746
P39060	COL18A1	Collagen, type XVIII, alpha 1	5.397	3.806	7.418	7.192	2.107	0.310	2.916	1.534
P07585	DCN	Decorin	6.765	7.430	3.766	3.543	2.325	1.418	1.380	0.429
Q02413	DSG1	Desmoglein-1	3.698	1.491	1.524	0.708	4.249	2.763	1.335	0.116
Q14956	GPNMB	Transmembrane glycoprotein NMB	1.086	0.477	2.759	0.782	1.122	0.718	3.195	2.869
P23229	ITGA6	Integrin alpha-6	1.153	0.404	1.049	0.137	-1.456	0.489	-1.742	0.325
P05556	ITGB1	Integrin beta-1	1.093	0.395	-1.091	0.307	1.316	0.656	1.064	0.401
P16144	ITGB4	Integrin beta-4	1.005	0.112	1.090	0.360	-1.025	0.744	1.261	1.419
Q07954	LRP1	Prolow-density lipoprotein receptor-related protein 1	1.045	0.189	-1.355	0.304	1.785	1.231	1.401	1.166

P35579	MYH9	Myosin, heavy chain 9, non-muscle	-4.227	0.046	-4.136	0.063	-3.408	0.121	-3.455	0.133
Q53GU8	TGFBI	Transforming growth factor, beta-induced	4.844	2.911	1.965	0.700	3.430	1.181	1.483	0.601
O60635	TSPAN1	Tetraspanin-1	2.935	2.343	4.539	1.591	-1.408	0.158	3.620	1.455
Immune response			**Mean**	**StDev**	**Mean**	**StDev**	**Mean**	**StDev**	**Mean**	**StDev**
B4DPJ2	ANXA11	Annexin A11	1.216	0.298	-1.046	0.220	1.340	0.883	1.007	0.483
P51572	BCAP31	B-cell receptor-associated protein 31	2.280	0.489	1.957	0.476	2.392	0.816	2.031	0.677
Q07021	C1QBP	Complement component 1 Q subcomponent-binding protein, mitochondrial	1.179	0.463	1.348	0.738	1.466	0.448	1.640	0.564
Q14UF6	CD55	Decay-accelerating factor splicing variant 1	6.439	10.734	5.406	9.488	2.115	1.659	1.637	1.520
P10909	CLU	Clusterin	3.584	1.284	2.726	1.009	3.652	0.970	2.796	0.837
P53634	CTSC	Cathepsin C	1.166	0.349	-1.270	0.374	1.415	0.320	-1.074	0.322
P02794	FTH1	Ferritin heavy chain	1.516	0.462	1.011	0.409	1.725	0.498	1.258	0.937
P06744	GPI	Glucose-6-phosphate isomerase	-1.205	0.176	-1.199	0.368	1.141	0.727	1.015	0.593
P10809	HSPD1	60 kDa heat shock protein, mitochondrial	-1.530	0.061	-1.463	0.101	-1.332	0.145	-1.266	0.286
P01876	IGHA1	Ig alpha-1 chain C region	-1.204	0.128	2.556	1.471	1.856	1.435	3.899	1.370
P0CG05	IGLC2	Ig lambda-2 chain C regions	3.805	2.079	4.361	2.833	3.551	2.652	3.503	2.956
Q12905	ILF2	Interleukin enhancer-binding factor 2	1.025	0.648	-1.026	0.560	-1.249	0.191	-1.313	0.072
P14174	MIF	Macrophage migration inhibitory factor	1.669	0.390	1.910	0.576	2.550	1.694	3.175	2.660
Q06830	PRDX1	Peroxiredoxin-1	-1.037	0.146	-1.108	0.173	1.177	0.218	1.077	0.149
P32119	PRDX2	Peroxiredoxin-2	-1.483	0.168	-1.277	0.357	-1.120	0.422	1.063	0.748
D3YTI9	TAPBP	TAPBP protein	1.584	0.935	1.240	0.371	1.345	1.148	-1.011	0.596
P63104	YWHAZ	14-3-3 protein zeta/delta	-1.046	0.181	-1.016	0.164	-1.234	0.204	-1.235	0.089
S100A proteins			**Mean**	**StDev**	**Mean**	**StDev**	**Mean**	**StDev**	**Mean**	**StDev**
P26447	S100A4	Protein S100-A4	7.593	3.718	6.884	1.880	9.307	3.698	8.669	1.764
P06703	S100A6	Protein S100-A6	2.144	0.955	1.779	0.584	2.336	1.104	1.899	0.759
P05109	S100A8	Protein S100-A8	-3.264	0.191	5.313	3.444	-8.236	0.105	1.685	1.431
P06702	S100A9	Protein S100-A9	-2.029	0.328	2.867	1.800	-1.232	1.572	2.540	2.727
P60903	S100A10	Protein S100-A10	3.474	0.550	2.921	0.806	3.182	0.969	2.994	1.487
P31949	S100A11	Protein S100-A11	1.206	0.546	1.624	0.684	1.322	1.570	1.582	1.443
Q9HCY8	S100A14	Protein S100-A14	-2.127	0.174	-1.425	0.135	-1.989	0.286	-1.190	0.668
Q96FQ6	S100A16	Protein S100-A16	1.479	0.756	4.813	2.812	2.263	1.822	5.803	2.845
Antioxidants			**Mean**	**StDev**	**Mean**	**StDev**	**Mean**	**StDev**	**Mean**	**StDev**
B4DSY2	GSTK1	Glutathione S-transferase kappa 1	1.227	0.207	1.034	0.048	-2.282	0.209	-2.618	0.265
Q06830	PRDX1	Peroxiredoxin-1	-1.037	0.146	-1.108	0.173	1.177	0.218	1.077	0.149
P32119	PRDX2	Peroxiredoxin-2	-1.483	0.168	-1.277	0.357	-1.120	0.422	1.063	0.748
P30048	PRDX3	Thioredoxin-dependent peroxide reductase, mitochondrial	-1.519	0.109	-1.145	0.234	1.187	0.377	1.608	0.785
P30044	PRDX5	Peroxiredoxin-5, mitochondrial	2.405	0.213	2.286	0.306	1.854	0.732	1.719	0.561
P30041	PRDX6	Peroxiredoxin-6	-1.657	0.166	-1.549	0.111	-2.672	0.068	-2.273	0.268
O60613	SEP15	15 kDa selenoprotein	4.500	3.579	5.490	1.893	5.154	4.948	5.312	0.463
P00441	SOD1	Superoxide dismutase [Cu-Zn]	1.080	0.570	1.253	0.782	1.126	0.335	1.270	0.518
P04179	SOD2	Superoxide dismutase 2	-1.203	0.236	1.959	1.288	1.135	0.409	2.303	0.948
Angiogenesis			**Mean**	**StDev**	**Mean**	**StDev**	**Mean**	**StDev**	**Mean**	**StDev**
P07355	ANXA2	Annexin A2	1.755	0.137	1.491	0.576	1.236	0.045	1.033	0.392
P06576	ATP5B	ATP synthase, H+ transporting, mitochondrial F1 complex, beta polypeptide	2.219	0.247	2.298	0.747	1.952	0.302	1.969	0.443
Q59E85	CAV1	Caveolin 1	-1.607	0.215	-1.113	0.086	-2.818	N/A	-2.671	N/A
P55290	CDH13	Cadherin 13	1.494	0.429	1.845	0.676	11.295	15.135	12.714	16.914

P39060	COL18A1	Collagen, type XVIII, alpha 1	5.397	3.806	7.418	7.192	2.107	0.310	2.916	1.534
P06744	GPI	Glucose phosphate isomerase	-1.205	0.176	-1.199	0.368	1.141	0.727	1.015	0.593
P35579	MYH9	Myosin, heavy chain 9, non-muscle	-4.227	0.046	-4.136	0.063	-3.408	0.121	-3.455	0.133
P19338	NCL	Nucleolin	-1.809	0.057	-1.505	0.179	-1.108	0.278	1.020	0.312
P13489	RNH1	Ribonuclease/angiogenin inhibitor 1	-1.130	0.283	1.406	0.538	-1.706	0.042	-1.127	0.019
Q9NQC3	RTN4	Reticulon 4	1.424	0.940	1.202	0.762	1.664	1.132	1.379	0.908

Supplementary Table S4. Corneal and limbal markers. Expression differences in primary CECs and LECs compared to hESC-LESCs and hiPSC-LESCs, presented as mean fold changes and standard deviation. Related to Figure 6.

UniProt	Protein symbol	Full name	CEC : hESC-LESCs		LEC : hESC-LESCs		CEC : hiPSC-LESCs		LEC : hiPSC-LESCs	
			Mean	StDev	Mean	StDev	Mean	StDev	Mean	StDev
P30838	ALDH3A1	Aldehyde dehydrogenase, dimeric NADP-preferring	14.003	8.006	9.766	3.903	15.458	10.817	10.275	4.730
P12830	CDH1	Cadherin 1, E-cadherin (epithelial)	1.782	0.545	1.903	0.431	1.697	0.447	1.760	0.188
P07585	DCN	Decorin	6.765	7.430	3.766	3.543	2.325	1.418	1.380	0.429
P06733	ENO1	Alpha-enolase	2.789	0.760	2.680	0.759	3.333	1.638	2.882	0.307
P08107	HSP70	Heat shock 70 kDa protein 1A/1B	2.235	0.640	2.467	0.287	2.095	0.606	2.248	0.268
P23229	ITGA6	Integrin alpha-6	1.153	0.404	1.049	0.137	-1.456	0.489	-1.742	0.325
P05556	ITGB1	Integrin beta-1	1.093	0.395	-1.091	0.307	1.316	0.656	1.064	0.401
P16144	ITGB4	Integrin beta-4	1.005	0.112	1.090	0.360	-1.025	0.744	1.261	1.419
P12035	KRT3	Keratin, type II cytoskeletal 3	6.121	2.465	-1.701	0.337	10.922	2.590	-1.424	0.401
P13647	KRT5	Keratin, type II cytoskeletal 5	2.536	2.378	-1.852	0.290	2.068	1.917	-2.448	0.257
P08729	KRT7	Keratin, type II cytoskeletal 7	-7.534	0.096	-7.395	0.084	-14.231	0.056	-17.539	0.027
P05787	KRT8	Keratin, type II cytoskeletal 8	-12.284	0.058	-13.854	0.035	-16.480	0.027	-18.166	0.013
Q99456	KRT12	Keratin, type I cytoskeletal 12	5.642	2.373	1.362	1.145	5.673	3.631	1.075	0.661
P02533	KRT14	Keratin, type I cytoskeletal 14	1.163	0.267	-2.056	0.296	-1.186	0.217	-2.901	0.219
P08727	KRT19	Keratin, type I cytoskeletal 19	-6.246	0.082	-4.112	0.112	-14.013	0.056	-10.301	0.071
Q8WXI7	MUC16	Mucin 16	8.146	0.989	3.761	0.404	6.384	N/A	4.438	N/A
P01011	SERPINA3	Alpha-1-antichymotrypsin	-1.013	N/A	-1.005	N/A	-1.760	0.078	-1.909	0.152
P00441	SOD1	Superoxide dismutase [Cu-Zn]	1.080	0.570	1.253	0.782	1.126	0.335	1.270	0.518
P04179	SOD2	Superoxide dismutase 2	-1.203	0.236	1.959	1.288	1.135	0.409	2.303	0.948
Q15582	TGFBI	Transforming growth factor-beta-induced protein ig-h3	4.844	2.911	1.965	0.700	3.430	1.181	1.483	0.601
P08670	VIM	Vimnetin	-8.863	0.077	-2.943	0.104	-5.338	0.136	-1.892	0.181

Supplementary Table S5. Top 55 most abundant proteins in human corneal epithelium. Expression differences in primary CECs and LECs compared to hESC-LESCs and hiPSC-LESCs, presented as mean fold changes and standard deviation. Out of the 55 proteins identified by Dyrlund *et al.*, 44 were present in our data set.

#	UniProt	Protein symbol	Full name	CEC : hESC-LESCs		LEC : hESC-LESCs		CEC : hiPSC-LESCs		LEC : hiPSC-LESCs	
				Mean	StDev	Mean	StDev	Mean	StDev	Mean	StDev
1	Q99456	KRT12	Keratin, type I cytoskeletal 12	5.642	2.373	1.362	1.145	5.673	3.631	1.075	0.661
2	P13647	KRT5	Keratin, type II cytoskeletal 5	2.536	2.378	-1.852	0.290	2.068	1.917	-2.448	0.257
3	P02538	KRT6A	Keratin, type II cytoskeletal 6A	N/A	N/A	N/A	N/A	N/A	N/A	N/A	N/A
4	P12035	KRT3	Keratin, type II cytoskeletal 3	6.121	2.465	-1.701	0.337	10.922	2.590	-1.424	0.401
5	P35908	KRT2	Keratin, type II cytoskeletal 2 oral	N/A	N/A	N/A	N/A	N/A	N/A	N/A	N/A
6	P19012	KRT15	Keratin, type I cytoskeletal 15	N/A	N/A	N/A	N/A	N/A	N/A	N/A	N/A
7	P19013	KRT4	Keratin, type II cytoskeletal 4	1.870	1.504	1.519	0.973	1.581	0.439	1.336	0.072
8	P08727	KRT19	Keratin, type I cytoskeletal 19	-6.246	0.082	-4.112	0.112	-14.013	0.056	-10.301	0.071
9	P30838	ALDH3A1	Aldehyde dehydrogenase, dimeric NADP-preferring	14.003	8.006	9.766	3.903	15.458	10.817	10.275	4.730
10	P06733	ENO1	Alpha-enolase	2.789	0.760	2.680	0.759	3.333	1.638	2.882	0.307
11	P02533	KRT14	Keratin, type I cytoskeletal 14	1.163	0.267	-2.056	0.296	-1.186	0.217	-2.901	0.219
12	P05787	KRT8	Keratin, type II cytoskeletal 8	-12.284	0.058	-13.854	0.035	-16.480	0.027	-18.166	0.013
13	P62805	HIST1H4A	Histone H4	1.213	0.482	1.511	0.380	1.440	0.177	1.809	0.215
14	P06899	HIST1H2BJ	Histone H2B type 1-C/E/F/G/I	3.589	2.191	3.589	2.191	3.589	2.191	3.589	2.191
15	P29401	TKT	Transketolase	3.740	0.943	2.506	1.173	3.805	0.921	2.572	1.102
16	P60709	ACTB	Actin, cytoplasmic 1	N/A	N/A	N/A	N/A	N/A	N/A	N/A	N/A
17	A8K9J7	HISTH2B	Histone H2B type 1-B	2.204	1.529	2.303	1.219	2.393	0.626	2.551	0.457
18	P02452	COL1A1	Collagen alpha-1(I) chain	N/A	N/A	N/A	N/A	N/A	N/A	N/A	N/A
19	P07355	ANXA2	Annexin A2	1.755	0.137	1.491	0.576	1.236	0.045	1.033	0.392
20	P04792	HSPB1	Heat shock protein beta-1	-1.530	0.061	-1.463	0.101	-1.332	0.145	-1.266	0.286
21	Q15582	TGFBI	Transforming growth factor-beta-induced protein ig-h3	4.844	2.911	1.965	0.700	3.430	1.181	1.483	0.601
22	P02768	ALB	Serum albumin	-1.559	0.099	1.180	0.330	-2.718	0.044	-1.406	0.294
23	P13646	KRT13	Keratin, type I cytoskeletal 13	N/A	N/A	N/A	N/A	N/A	N/A	N/A	N/A
24	P04406	GAPDH	Glyceraldehyde-3-phosphate dehydrogenase	-1.014	0.118	-1.073	0.346	-1.157	0.078	-1.204	0.323
25	B7Z5E3	LDHA	L-lactate dehydrogenase A chain	1.794	0.763	1.679	0.685	1.970	0.360	1.925	0.672
26	P26447	S100A4	Protein S100-A4	7.593	3.718	6.884	1.880	9.307	3.698	8.669	1.764
27	P14618	PKM2	Pyruvate kinase isozymes M1/M2	-1.097	0.128	-1.054	0.314	1.007	0.233	1.069	0.574
28	P00352	ALDH1A1	Retinal dehydrogenase 1	4.057	0.916	4.261	0.649	3.749	1.056	3.853	0.548
29	P68104	EEF1A1	Elongation factor 1-alpha 1	-2.777	0.033	-2.777	0.094	-2.556	0.048	-2.561	0.124
30	P02794	FTH1	Ferritin heavy chain	1.516	0.462	1.011	0.409	1.725	0.498	1.258	0.937
31	A8MUB1	TUBA4A	Tubulin alpha-4A chain	2.904	1.967	2.454	1.177	1.280	0.610	1.098	0.719
32	P31947	SFN	Stratifin 14-3-3 protein sigma	-1.144	0.227	-1.031	0.398	-1.175	0.169	-1.126	0.172
33	Q06830	PRDX1	Peroxiredoxin-1	-1.037	0.146	-1.108	0.173	1.177	0.218	1.077	0.149
34	P05783	KRT18	Keratin, type I cytoskeletal 18	-3.116	0.115	-5.627	0.014	-3.882	0.135	-7.454	0.010
35	P09211	GSTP1	Glutathione S-transferase P	-1.687	0.285	-2.017	0.134	-1.499	0.279	-1.814	0.115
36	P06396	GSN	Gelsolin	2.291	0.613	2.078	0.152	2.227	0.460	2.000	0.325
37	P08123	COL1A2	Collagen alpha-2(I) chain	N/A	N/A	N/A	N/A	N/A	N/A	N/A	N/A
38	P08107	HSP70	Heat shock 70 kDa protein 1A/1B	2.235	0.640	2.467	0.287	2.095	0.606	2.248	0.268
39	P63104	YWHAZ	14-3-3 protein zeta/delta	-1.046	0.181	-1.016	0.164	-1.234	0.204	-1.235	0.089
40	P01857	IGHG1	Ig gamma-1 chain C region	N/A	N/A	N/A	N/A	N/A	N/A	N/A	N/A

41	P04264	KRT1	Keratin, type II cytoskeletal 1	1.607	0.091	-4.088	0.018	3.577	0.227	-1.856	0.026
42	B4E0A4	COL6A3	Collagen alpha-3(VI) chain	-1.024	N/A	3.925	N/A	1.103	N/A	4.330	N/A
43	P31946	YWHAB	14-3-3 protein beta/alpha	1.109	0.091	1.034	0.544	1.106	0.564	-1.020	0.509
44	P61981	YWHAG	14-3-3 protein gamma	1.105	0.720	-1.050	0.595	-1.045	0.639	-1.247	0.437
45	P27348	YWHAQ	14-3-3 protein theta	-1.866	0.301	-1.576	0.340	-3.657	0.074	-2.723	0.105
46	P62258	YWHAE	14-3-3 protein epsilon	-1.181	0.266	-1.223	0.156	1.226	0.499	1.079	0.401
47	P68371	TUBB2C	Tubulin beta-2C chain	-1.155	0.193	-1.150	0.230	-1.013	0.402	-1.055	0.413
48	Q5JP53	TUBB	Tubulin, beta	-1.776	0.162	-1.532	0.199	-1.615	0.212	-1.405	0.306
49	B4DLR8	NQO1	NAD(P)H dehydrogenase, quinone 1	1.308	0.394	1.077	0.457	2.080	1.629	1.518	0.932
50	Q13885	TUBB2A	Tubulin beta-2A chain	N/A	N/A	N/A	N/A	N/A	N/A	N/A	N/A
51	P11142	HSPA8	Heat shock cognate 71 kDa protein	-2.562	0.105	-2.767	0.041	-1.865	0.179	-2.086	0.081
52	P31949	S100A11	Protein S100-A11	1.206	0.546	1.624	0.684	1.322	1.570	1.582	1.443
53	P01834	IGKC	Ig kappa chain C region	N/A	N/A	N/A	N/A	N/A	N/A	N/A	N/A
54	Q15149	PLEC	Plectin	1.039	0.192	1.406	1.074	-1.010	0.323	1.903	2.119
55	P30044	PRDX5	Peroxiredoxin-5, mitochondrial	2.405	0.213	2.286	0.306	1.854	0.732	1.719	0.561
